# *Wnt10b* Deficiency Results in Age-Dependent Loss of Bone Mass and Progressive Reduction of Mesenchymal Progenitor Cells

**DOI:** 10.1002/jbmr.118

**Published:** 2010-04-30

**Authors:** Jennifer R Stevens, Gustavo A Miranda-Carboni, Meredith A Singer, Sean M Brugger, Karen M Lyons, Timothy F Lane

**Affiliations:** 1 Biological Chemistry, David Geffen School of Medicine, University of California Los Angeles Los Angeles, CA, USA; 2 Department Obstetrics and Gynecology, David Geffen School of Medicine, University of California Los Angeles Los Angeles, CA, USA; 3 Department of Orthopaedic Surgery, David Geffen School of Medicine, University of California Los Angeles Los Angeles, CA, USA; 4 Molecular Biology Institute, David Geffen School of Medicine, University of California Los Angeles Los Angeles, CA, USA; 5 Department Molecular, Cellular and Developmental Biology, David Geffen School of Medicine, University of California Los Angeles Los Angeles, CA, USA; 6 Jonsson Comprehensive Cancer Center, David Geffen School of Medicine, University of California Los Angeles Los Angeles, CA, USA

**Keywords:** Wnt Signaling, osteoporosis, Haploinsufficiency, mesenchymal progenitor cell (MPC)

## Abstract

*Wnt10b* is a canonical Wnt ligand expressed in developing bone and has been linked to mesenchymal progenitor functions in mice and humans. Because Wnt signaling has been shown to play an important role in progenitor maintenance in a variety of adult tissues, we examined bone deposition and growth rates throughout postnatal development in *Wnt10b*-null mice. Using bone histomorphometry and micro–computed tomographic (µCT) studies, we demonstrate that trabecular bone deposition is slightly enhanced in *Wnt10b*-null mice at 1 month of age, followed by progressive loss with age. Importantly, we find that *Wnt10b* is required for maintenance of adult bone density in multiple backgrounds of inbred mice and that both copies of the *Wnt10b* gene are required to maintain normal bone density in 6-month-old animals. We go on to show that the loss in trabecular bone in *Wnt10b*-null mice is associated with a reduction in the number of bone marrow–derived mesenchymal progenitors (MPCs) using in vitro colony-forming unit assays and marker analysis. Analysis of osteogenic gene expression in primary bone marrow stromal cells demonstrated reductions in expression of several osteoblast differentiation markers. Taken together, our results indicate that *Wnt10b* is uniquely required for maintenance of mesenchymal progenitor activity in adult bone. The results show the significance of studying individual Wnt ligands and their potentially unique contribution in the context of aging and disease. © 2010 American Society for Bone and Mineral Research.

## Introduction

During embryonic development, mesenchymal progenitor cells (MPCs) contribute to the various derivatives of mesenchyme, including bone, fat, muscle, and cartilage. In adults, cells with MPC-like characteristics are present in most tissues and may contribute to remodeling and repair of mesenchymal derivatives.(1,2) Wnt signaling has been implicated in various aspects of mesenchymal development,(3) including suggested roles in the maintenance, proliferation, and differentiation of postembryonic bone(4–6) and in establishment and maintenance of adult mesenchymal stem cells.(7) Misregulation of the Wnt pathway also has been noted in osteosarcoma(8) and in other disorders of mesenchymal origin. While not excluding other mesenchymal derivatives, activation of Wnt signaling generally is associated with expansion of the osteoblast and chondrocyte lineages in vivo and in vitro,(3,9–12) and canonical Wnt ligands lead to expansion of MPCs at the expense of differentiation in vitro.(13,14)

*Wnt* genes encode a family of conserved extracellular growth factors, with 19 members in mammals. Most Wnt proteins are thought to act as ligands for cell surface receptor complexes composed of frizzled (Fz) and low-density lipoprotein (LDL)–receptor–related protein 5/6 (LRP5/6) family members. Downstream of Fz-LRP5/6 complexes, canonical Wnt signaling results in stabilization and translocation of β-catenin to the nucleus, where it binds to T-cell factor/lymphoid enhancer factor (TCF) Lef transcription factors. β-Catenin–TCF/Lef complexes activate transcription of a variety of Wnt-responsive genes, including genes involved in proliferation and osteoblastogenesis.(15)

*Wnt10b* is expressed in the bone marrow, postnatal growth plate,(16) osteoblastic precursors,(17) and various other stem cell compartments. It has been shown to activate transcription of canonical Wnt targets, including *Id2* and *cyclin D1.*(18,19) Previous work has shown that transgenic overexpression of *Wnt10b* in mesenchymal derivatives leads to increased bone density, increased trabecular number and thickness in vivo, and accelerated osteoblastogenesis in vitro.(19,20) This work also demonstrated that *Wnt10b*-null mice have reduced trabecular mass at 8 weeks; however, a full characterization of the observed phenotype was not undertaken.

To elucidate cellular defects contributing to low adult bone mass in *Wnt10b-*null mice, we examined bone deposition and growth rates throughout postnatal development. We found that *Wnt10b*-null mice display a progressive loss of trabecular structure and went on to identify a pronounced trabecular phenotype in *Wnt10b* heterozygous mice. Interestingly, we found that *Wnt10b*-null animals showed enhanced trabecular structure at 2 and 4 weeks of age but that the increase was rapidly followed by progressive osteopenia from 2 to 6 months of age. These results lead us to hypothesize that *Wnt10b* expression helps to maintain osteoblast progenitors in an undifferentiated state and that loss of *Wnt10b* expression results in either increased differentiation or decreased self-renewal of mesenchymal progenitors. The result of decreased *Wnt10b* expression thus could lead to early exhaustion of the progenitor pool and subsequent loss of bone mass with age. Consistent with this hypothesis, we find that the age-progressive osteopenia is associated with decreased recovery and activity of mesenchymal progenitors from bone marrow. Specifically, the loss of *Wnt10b* resulted in decreased recovery of mesenchymal progenitor cells (MPCs) and MPC lineage–derived osteoblastic activity as assayed by in vitro colony-forming unit (CFU) assays and marker analyses. Taken together, these results suggest that *Wnt10b* is a key regulator of the mesenchymal progenitor fate and that it is required late in life for maintenance of postnatal osteogenic progenitors.

## Materials and Methods

### Generation and genotyping of *Wnt10b*-null mice

**Fig. 1:**
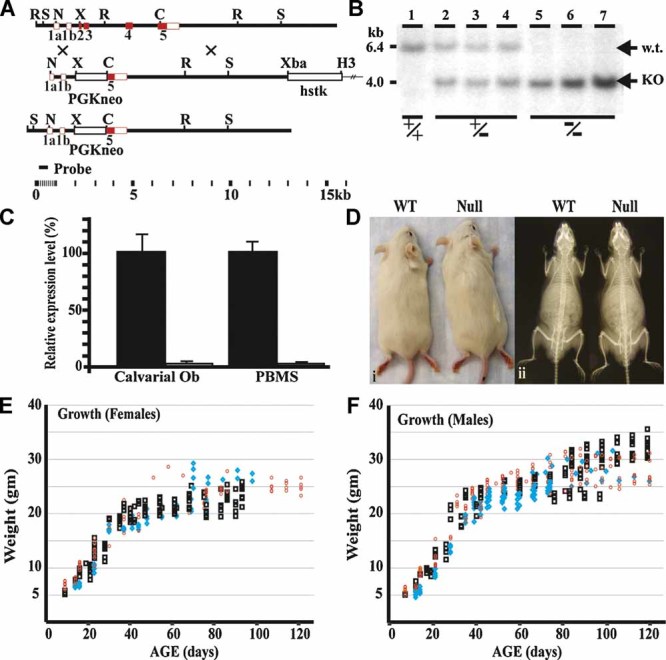
*Wnt10b*-null mice appear morphologically normal and have normal growth rates. (*A*) The design of the *Wnt10b*^Δex2-5^ targeting vector is illustrated. The targeting vector completely eliminates the coding region between exon 2 and the *Cla-1* site in exon 5. The construct was transfected into mouse ES^TC1^ cells, and clones were selected for homologous recombination. Three targeted clones were identified showing the expected integration within the *Wnt10b* locus. (*B*) Southern blot analysis of genomic DNA isolated from wild-type (WT), heterozygous, and *Wnt10b*-null mice. DNA was obtained from a litter of pups created from a cross between *Wnt10b*^Δex2-5^-heterozygous parents. DNA was digested with both *Sac-1* (S) and *Cla-1* (C) and hybridized with a probe to sequences 5' to the construct (Probe). The expected 2.4-kb shift is observed. (*C*) Quantitative PCR analysis of *Wnt10b* mRNA expression in calvarial osteoblasts and primary bone marrow stromal cells (PBMSCs) isolated from WT and *Wnt10b*-null mice. (*D*) Images of WT and *Wnt10b*-null mice showing no difference in overall gross morphology when examining either the exterior (i) or X-ray images of skeletal structures (ii). (*E, F*) Graph of growth rate for female (*E*) and male (*F*) WT (*black box*), heterozygous (*blue diamond*), and *Wnt10b*-null (*red circle*) mice. All experiments in this figure use mice in the FVB background.

**Fig. 3:**
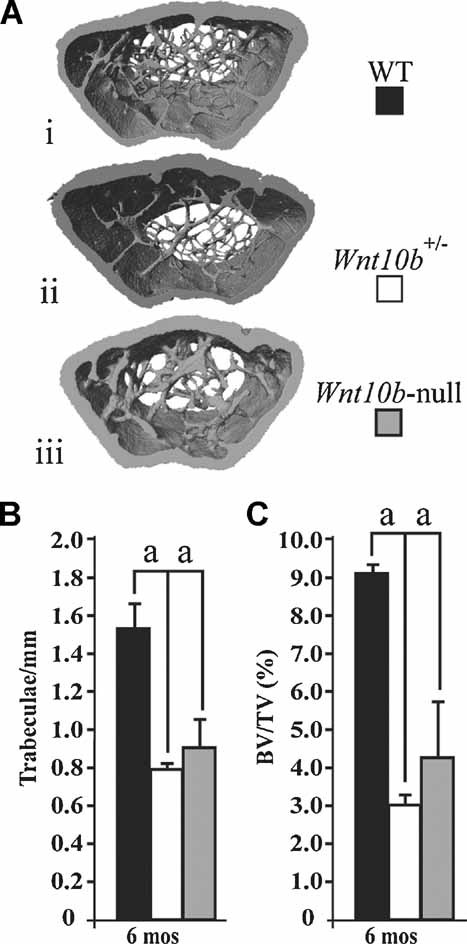
*Wnt10b* is also required for maintenance of bone density in the C57/BL6 strain of mice, and loss of one allele is sufficient to generate severe osteopenia by 6 months of age. (*A*) µCT images of distal femurs from WT (i), *Wnt10b*^+/–^ (ii), and *Wnt10b*-null (iii) mice. (*A–C*) 3D µCT analysis of 6-month-old WT (*n* = 4, *black bar*), *Wnt10b*^+/–^ (*n* = 4, *white bar*), and *Wnt10b*-null (*n* = 4, *black bar*) mice for trabeculae/mm (*B*) and bone volume fraction (BV/TV%, *C*). All experiments in this figure use mice in the C57/Bl6 background. Statistical significance was evaluated by Student's *t* test; ^a^*p* < .05.

**Fig. 5:**
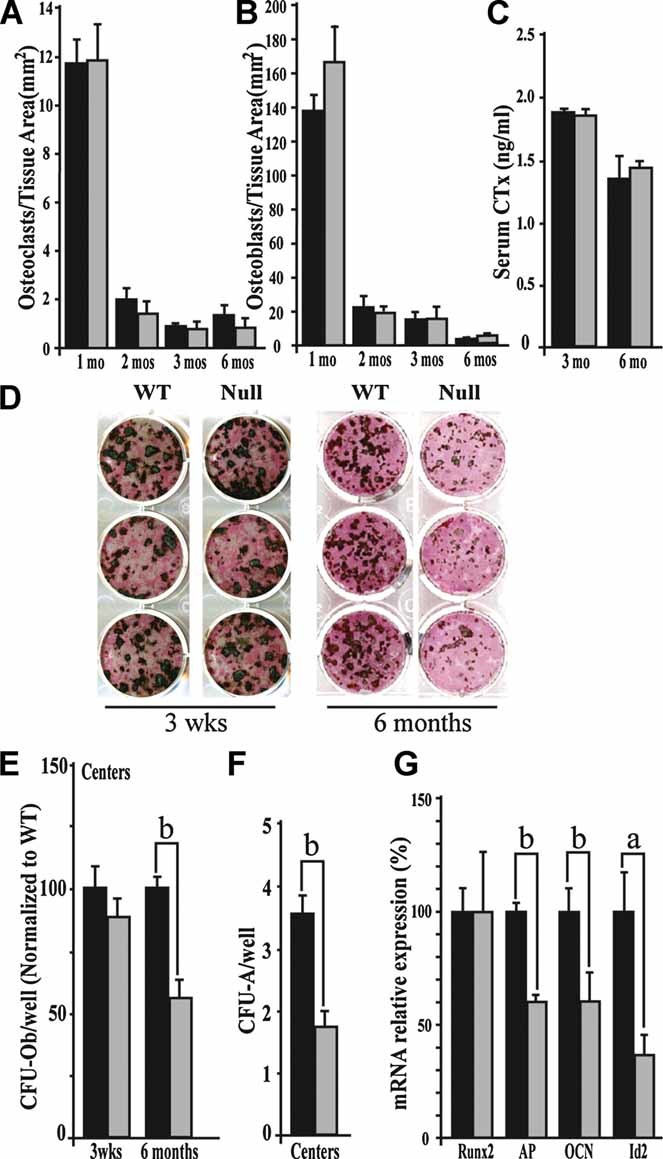
The age-progressive osteopenia in *Wnt10b*-null mice is not due to abnormal bone resorption but is associated with decreased numbers of osteogenic and adipogenic progenitor cells in aged mice. Histomorphometric analysis shows no significant difference in the number of osteoclasts (*A*) or osteoblasts (*B*) at 1 (*n* = 4), 2 (*n* = 7), 3 (*n* = 7), and 6 (*n* = 7) months of age. Analysis of serum CTx (*C*) in 3- (*n* = 3) and 6-month-old (*n* = 3) animals shows no significant difference in osteoclast activity. (*D*) Dual alkaline phosphatase/von Kossa stains of 3-week- and 6-month-old primary bone marrow stromal cells (PBMSCs) induced to differentiate down the osteoblastic lineage show a reduction in the number of ossified centers in *Wnt10b*-null PBMSCs at 6 months but no difference at 3 weeks. (*E*) *Wnt10b-*null PBMSC (*gray bar*) shows a statistically significant decrease in the number of centers compared with WT (*black bar*) at 6 months but not 3 weeks. (*F*) *Wnt10b-*null PBMSC (*gray bar*) shows a statistically significant decrease in the number of adipogenic centers at 6 months compared with WT (*black bar*). (*G*) Quantitative PCR analysis of mRNA isolated from WT (*black bar*) and *Wnt10b*-null (*gray bar*) PBMSCs following 10-day expansion in growth medium. *Wnt10b*-null PBMSCs from 6-month-old mice show decreases in *alkaline phosphatase* (*AP*), *osteocalcin* (*OCN*), and *Id2* expression but no change in *Runx2*. All experiments in this figure were performed in triplicate. Technical replicates from a representative biologic replicate are shown. All experiments in this figure use mice in the FVB background, with the exception of the CFU-A experiments, in which C57/Bl6 mice were used. Statistical significance was evaluated by Student's *t* test; ^a^*p* < .05; ^b^*p* < 01.

A null allele (*Wnt10b*^Δex2-5^) was generated through homologous recombination by introducing the neomycin resistance gene in lieu of the endogenous exons 2 to 5 of the mouse *Wnt10b* gene, including most of intron 4 and part of exon 5. The *Wnt10b*^Δex2-5^ targeting vector (Fig. 1*A*) was created from 129^SvEv^ genomic fragments cloned into the pPNT targeting vector.(21) The vector then was electroporated into TC-1 embryonic stem cells,(22) and colonies were selected by growth on embryonic fibroblasts in medium supplemented with LIF-1, G418, and FIAU. Three properly targeted colonies were identified by Southern blot analysis and used to generate chimeric founder animals. Founders were backcrossed into FVB/N or C57Bl6 for 12 generations to create *Wnt10b*^Δ2-5FVB^ and *Wnt10b*^Δex2-5Bl6^, respectively. Genotyping of F_1_ through F_9_ animals was performed by genomic Southern blot, and PCR was used thereafter. Genomic DNA was prepared from tail-snip biopsies following standard protocols. Southern blot analysis was carried out on *Sal1-Cla1* digested DNA, and blots were probed with 5' genomic fragment (Fig. 1*A*) not included in the original targeting vector. Once lines were established, they were monitored by PCR using a combination of neospecific and exon 5–specific primers. PCR conditions and primer sequences can be found in Supplemental Table 1 and Supplemental Materials and Methods. Except for Fig. 3 and one panel of Fig. 5, all studies presented in this article were completed using mice in the FVB background.

### Quantitative RT-PCR

Total RNA was extracted from plastic-adherent primary bone marrow–derived stromal cells and passage 2 calvarial osteoblasts using TRIzol (Invitrogen, Carlsbad, CA, USA) following the manufacturer's instructions. RNA was DNaseI treated and cDNA generated with the iScript cDNA Synthesis Kit (BioRad, Hercules, CA, USA) following the manufacturer's instructions. Quantitative RT-PCR was performed on cDNA using the Power SYBR Green amplification system (Applied Biosystems, Carlsbad, CA, USA) following the manufacturer's instructions. PCR conditions and primer sequences can be found in Supplemental Table 2 and Supplemental Materials and Methods.

### Histomorphometry and micro–computed tomography (µCT)

One-, two-, three-, and six-month-old wild-type (WT) and *Wnt10b-*null male mice were given single intraperitoneal injections of 20 mg of calcein (C0875, Sigma Aldrich, St. Louis, MO, USA) per kilogram of body weight on day 1 and 20 mg of demeclocycline (D6140, Sigma Aldrich) per kilogram of body weight on day 3 (1 month), day 6 (2 months), or day 8 (3 and 6 months). Mice were euthanized 2 days following final injection. Femurs from each mouse were dissected free of tissue, fixed in 70% ethanol, and embedded in methyl methacrylate (right femur) or prepared for analysis by µCT (left femur). For histomorphometry, 5-µm-thick longitudinal sections were cut through the trabecular region of the distal femur and toluidine blue stained for analysis using image analysis software (Osteomeasure, Atlanta, GA, USA). Osteoblast and osteoclast numbers were measured, and the mineral apposition rate (MAR) and mineralizing surface/bone surface (MS/BS) were calculated as described previously.(23) For µCT, the left distal femur from 6-month-old male C57/Bl6 mice was scanned at 6-µm resolution. Images were generated using the 3D visualization/animation component of the SkyScan 1172 X-ray microtomograph and CT analyzer software (Kontich, Belgium).

### Primary bone marrow stromal cell isolation, osteo/adipogenic differentiation, and histologic staining

Tibias and femurs of WT and *Wnt10b*-null mice at 6 months of age were flushed with complete growth media (DMEM supplemented with 10% FBS; Omega Scientific, Tarzana, CA, USA), 5% l-glutamine, and 5% penicillin-streptomycin (CellGro, Lawrence, KS, USA) and passed through a 40-µm nylon mesh cell strainer (Becton Dickinson, Franklin Lakes, NJ, USA) to make a single-cell suspension. Total bone marrow was suspended in complete medium (for osteogenesis) or Mesencult (for adipogenesis) at 2 × 10^6^ nucleated cells per milliliter, seeded at 2 × 10^6^ nucleated cells per well in 12-well tissue culture plates, and incubated at 37°C in 5% CO_2_ for 5 days. Nonadherent cells then were removed, and adherent cells were washed, fed, and grown to 70% confluence in complete growth medium or Mesencult as indicated, at which point cells were switched to osteogenic medium (α-MEM supplemented with 10% FBS, 5% l-glutamine, 5% penicillin-streptomycin, and 2 mM β-glycerophosphate; G9891, Sigma Aldrich) and 50 µg/mL of l-ascorbic acid (A8960, Sigma Aldrich) or adipogenic medium (DMEM supplemented with 10% FBS, 5% l-glutamine, 5% penicillin-streptomycin, and 1 µM PPARγ agonist GW1929). Medium was replenished every third day until formation of osteogenic bone nodules (for osteogenesis assays) or formation of lipid droplets (for adipogenesis assays) in control wells. Colonies then were stained for alkaline phosphatase activity (86R, Sigma Aldrich) following the manufacturer's instructions and calcium deposition (nodulation) by incubation in 5% silver nitrate (S486, Fisher Scientific, Waltham, MA, USA) solution under ultraviolet (UV) light for 60 minutes or stained with oil red O following standard protocols. Nodulation was quantified as a direct count of the number of ossification centers present, and adipogenesis was assessed by enumeration of oil red O^+^ colonies.

### Enumeration and differentiation of mesenchymal stem cells harvested in Mesencult

Primary bone marrow stromal cells were isolated as described earlier. Total bone marrow was diluted in Mesencult basal medium plus supplements (Stem Cell Technologies, Vancouver, BC, Canada) and seeded at 5.0 × 10^5^ nucleated cells per well of a 6-well plate. Cells were maintained at 37°C in 5% CO_2_ for 10 days. Colonies were stained with Geimsa, and the colony-forming units fibroblastic (CFU-F) were enumerated by counting. For osteogenic and/or adipogenic differentiation, cells were switched to osteogenic or adipogenic medium (described earlier) at day 10, with subsequent medium changes every third day until formation of osteogenic nodules and/or formation of lipid droplets. The plates then were stained and enumerated by counting positive colonies.

### Serology

Blood from 3- and 6-month-old WT and *Wnt10b*-null mice was harvested at the time of euthanization, and serum was prepared. Serum cross-linked C-telopeptide (CTx) was measured using a RatLaps ELISA Kit (1RTL4000, Nordic Biosciences, Herlev, Denmark) following the manufacturer's instructions.

## Results

### *Wnt10b*-null mice appear morphologically normal and have normal growth rates

*Wnt10b-*null mice were generated by homologous recombination, replacing exons 2 to 4 and a portion of exon 5 with a neomycin resistance cassette (Fig. 1*A*). Deletion of the *Wnt10b* locus was validated by Southern blot analysis using a flanking probe (Fig. 1*B*). Quantitative RT-PCR of primary bone marrow stromal cells (PBMSCs) and calvarial osteoblasts demonstrates expression of *Wnt10b* mRNA in these cells and confirms that mice homozygous for deletion of the *Wnt10b* locus do not express *Wnt10b* mRNA (Fig. 1*C*). Outwardly, *Wnt10b-*null mice appear normal (Fig. 1*D*, i) and have normal litter size, and similar growth, as assessed by weekly weight measurements from birth to 4 months of age (Fig. 1*E, F*). Gross morphologic examination of the skeletal structure of *Wnt10b*-null mice reveals no apparent defects in either the length or width of the long bones (Fig. 1*D*, ii).

### *Wnt10b*-null mice exhibit enhanced early maturation followed by age-progressive loss of trabecular bone (osteopenia)

**Fig. 2:**
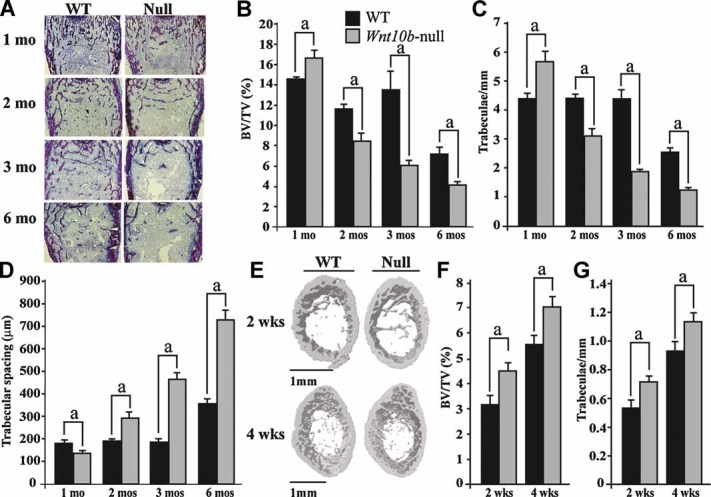
*Wnt10b*-null mice show increased bone accrual at young ages followed by an age-dependent loss of trabecular bone. (*A*) Toluidine blue–stained methyl methacrylate sections were prepared from the distal femurs of 1-, 2-, 3-, and 6-month-old WT and *Wnt10b*-null mice. (*B–D*) Morphometric properties of distal femurs from 1- (*n* = 4), 2- (*n* = 8), 3- (*n* = 7), and 6-month-old (*n* = 10) male WT (*black bar*) and *Wnt10b*-null (*gray bar*) mice. Femurs were analyzed for bone volume fraction (BV/TV%, *B*), trabeculae/mm (*C*), and trabecular spacing (*D*). (*E*) µCT images of distal femurs from 2- and 4-week-old WT and *Wnt10b*-null mice. (*F, G*) 3D µCT analysis of 1- (*n* = 5) and 4-week-old (*n* = 8) male (*black bar*) and *Wnt10b*-null (*gray bar*) mice for bone volume fraction (BV/TV%, *F*) and trabeculae/mm (*G*). All experiments in this figure use mice in the FVB background. Statistical significance was evaluated by Student's *t* test; ^a^*p* < .05.

Two-dimensional histomorphometry was carried out on the distal femoral metaphyses from 1-, 2-, 3-, and 6-month-old WT and *Wnt10b-*null mice backcrossed into the FVB background (Fig. 2*A*). One-month-old *Wnt10b-*null mice have statistically significant increases in bone volume fraction (BV/TV; Fig. 2*B*) and trabecular number (Tb.N; Fig. 2*C*) with concurrent decreases in trabecular spacing (Tb.Sp; Fig. 2*D*). WT mice show relatively little difference in all structural parameters between 1 and 3 months of age. At 6 months of age, WT mice show decreases in bone volume fraction and trabecular number with concurrent increases in trabecular spacing (Fig. 2*B–D*). In contrast, *Wnt10b-*null mice show progressive reductions in trabecular mass as they mature, indicating a loss of the ability to maintain trabecular mass. At 2, 3, and 6 months of age, *Wnt10b-*null mice show significant decreases in both bone volume fraction (BV/TV; Fig. 2*B*) and trabecular number (Fig. 2*C*) with concurrent increases in trabecular spacing (Fig. 2*D*). Analysis of trabecular and cortical thickness revealed no differences in these parameters at 1, 2, 3, or 6 months of age (data not shown). These results were confirmed independently by 3D µCT analyses (data not shown). Trabecular connectivity (Conn.D) measurements are consistent with measurements of trabecular number and spacing, showing no difference at 1 month of age but an age-dependent decrease beginning at 2 months of age (data not shown).

In order to further examine the interesting observation that structural parameters are increased at 1 months of age in *Wnt10b*-null mice, we performed additional µCT analyses on 2- and 4-week-old WT and *Wnt10b*-null mice (Fig. 2*E*). *Wnt10b*-null mice show an approximately 1.5-fold increase in bone volume fraction at 2 weeks and an approximately 1.2-fold increase at 4 weeks (BV/TV; Fig. 2*F*). These increases in BV/TV are associated with an increase in the number of trabeculae per millimeter (Fig. 2*G*) with no alteration in trabecular thickness (Fig. 2*G*; data not shown).

Additionally, µCT analysis of femurs from WT and *Wnt10b*-null mice backcrossed into the C57/Bl6 background line (Fig. 3*A–C*) also show a 50% decrease in bone volume fraction (BV/TV; Fig. 3*A, C*) and a 50% decrease in trabeculae per millimeter (Fig. 3*A, B*). In combination with the data presented in Fig. 1, these data illustrate that the bone-loss phenotype is robust and is present in at least two genetic backgrounds. Interestingly, this analysis also showed that mice heterozygous for deletion of the *Wnt10b* gene display a loss of trabecular bone at 6 months of age (Fig. 3*A*, ii, *B, C*). We have not determined whether bone loss is progressive in the C57 background, but these mice also grow at normal rates and do not appear to be different from FVB mice carrying the null allele.

**Fig. 4:**
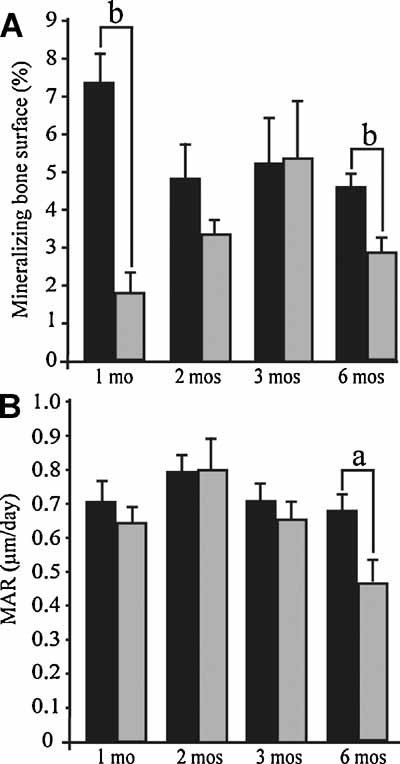
Dynamic properties of bone formation in WT and *Wnt10b*-null mice. (*A, B*) Dynamic properties of bone formation for 1- (*n* = 4), 2- (*n* = 6), 3- (*n* = 5), and 6-month-old (*n* = 8) male WT (*black bar*) and *Wnt10b*-null (*gray bar*) mice. Femurs were analyzed for mineralizing bone surface (MS/BS%, *A*) and matrix apposition rate (µm/day, *B*). All experiments in this figure use mice in the FVB background. Statistical significance was evaluated by Student's *t* test; ^a^*p* < .05; ^b^*p* < .01.

Measurements of dynamic bone formation in FVB mice reveal that the extent of mineralization in WT mice declines when rapid growth ceases at 2 months of age, at which point it plateaus and remains constant (MS/BS; Fig. 4*A*). This is in contrast to *Wnt10b*-null mice, in which the percentage of bone undergoing active mineralization is far below WT levels at 1 month of age. As a percentage of surface area, mineralization of *Wnt10b*-null bone becomes more similar to that of WT mice at 3 months of age but subsequently drops back below WT levels by 6 months. Interestingly, the matrix apposition rate (MAR), a measure of the rate of bone formation, is equivalent in 1-, 2-, and 3-month-old animals, with significant reductions in this parameter observed by 6 months of age (MAR; Fig. 4*B*).

These data, along with the observation that bone volume fraction and trabecular number are increased in *Wnt10b* mice at 2 and 4 weeks of age, suggest a role for *Wnt10b* in maintaining bone-forming osteoprogenitor cells in an undifferentiated state. The early increases in bone volume fraction and trabecular number in 2- and 4-week-old mice could reflect an initial period of accelerated differentiation of mesenchymal/osteoprogenitors resulting in depletion of a pool of stem cells that are not maintained over the life of the animal.

### Age-progressive osteopenia in *Wnt10b*-null mice is not due to abnormal bone resorption

Type I osteoporosis in humans typically is characterized by excess bone resorption in the absence of increased bone formation and occurs primarily in postmenopausal females when estrogen levels decline. However, non-estrogen-dependent osteoporosis phenotypes have been reported for both males and females in humans. The observation that *Wnt10b-*null animals have decreased bone volume by 2 months of age suggests that *Wnt10b-*null animals represent a model of osteoporosis. Histomorphometric data show no difference in osteoblast or osteoclast number per trabecular bone area (Fig. 5*A, B*) nor an increase in osteoclast activity, as assessed by analysis of serum CTx levels (Fig. 5*C*). These results indicate that the age-progressive osteopenia is not due to increased bone resorption but rather is the result of decreased bone deposition. In accordance with this idea, we observed reductions in percent mineralizing surface in 1-, 2-, and 6-month-old animals (MS/BS; Fig. 4*A*).

### Primary bone marrow stromal cells from *Wnt10b*-null mice have fewer osteoprogenitors by 6 months of age

Previous work has suggested that overexpression of *Wnt10b* can enhance bone deposition,(19,20) leading to the suggestion that *Wnt10b* acts to promote differentiation. However, our data suggest that *Wnt10b* is important to maintain mesenchymal/osteoprogenitors and that loss of expression leads to premature bone maturation, as evidenced by enhanced bone formation at 2 and 4 weeks of age that is accompanied by a subsequent inability to maintain trabecular bone. To examine whether loss of *Wnt10b* affects the number and function of osteoprogenitors, we performed in vitro osteogenesis assays. Primary bone marrow stromal cells (PBMSCs) containing both mesenchymal and osteoprogenitor cells were isolated from 3-week- and 6-month-old WT and *Wnt10b-*null mice. Isolated cells were expanded for 10 days to remove most hematopoetic derivatives and then induced to differentiate down the osteoblastic lineage by growth in osteogenic medium (containing 50 µg/mL of ascorbic acid and 2 mM β-glycerol phosphate). On formation of ossified bone nodules, colonies are stained for alkaline phosphatase activity and for Ca/PO_4_ deposition with von Kossa stain. Progenitor cell number was assessed by counting the number of ossification centers present [colony-forming units osteoblast (CFU-Ob)]. No difference was seen in the number of osteogenic centers at 3 weeks, but a 50% decrease in the number of osteogenic centers was found in cells isolated from bone marrow stroma of *Wnt10b-*null animals at 6 months of age (Fig. 5*D, E*).

Next, we examined expression of osteogenic genes in PBMSCs harvested from 6-month-old mice using quantitative RT-PCR analysis of mRNA and found a 1.5-fold reduction in expression of osteoblast differentiation markers alkaline phosphatase (ALP) and osteocalcin (OCN) in *Wnt10b* mutants (Fig. 5*G*). Additionally, we saw a 2-fold reduction in expression of the Wnt target gene *Id2* (Fig. 5*G*). No change was observed in the expression of *Runx2* (Fig. 5*G*), an osteoblast associated transcription factor known to be indispensable for osteoblast differentiation.(24–26)

Subsequently, we examined whether loss of *Wnt10b* expression affected only the osteogenic lineage or whether the deficiency was at the level of a more primitive mesenchymal progenitor with both osteogenic and adipogenic potential. Since FVB mice are highly resistant to diet-induced weight gain, we examined adipogenesis in PBMSCs isolated from 6-month-old male C57/Bl6 mice using in vitro adipogenesis assays. PBMSCs were isolated in Mesencult medium and induced to differentiate down the adipogenic lineage by addition of GW1929, a small-molecule agonist of peroxisomal proliferator-activated receptor-γ (PPARγ). On formation of lipid droplets, cultures were stained with oil red O for enumeration of colony-forming units adipocyte (CFU-A). Similar to the results seen for osteogenesis in the FVB background, a 50% decrease in the number of adipogenic centers was found in cells isolated from bone marrow stroma of C57/Bl6 *Wnt10b-*null animals at 6 months compared with WT controls (Fig. 5*F*).

In order to determine if the observed decreases in colony number were due to alterations in the proliferative capacity of cells isolated from *Wnt10b-*null animals, we examined growth rates of PBMSCs harvested from 6-month-old WT and *Wnt10b-*null mice during expansion in growth medium and found no difference in the rate of proliferation in vitro (Supplemental Fig. 1*B*). We also examined proliferation in vivo in the proliferative zone of the growth plate from postnatal day 4 mice. Immunohistochemistry to detect proliferating cell nuclear antigen (PCNA), a marker of proliferation, reveals no difference in the number of PCNA^+^ nuclei (Supplemental Fig. 1*A*).

### *Wnt10b*-null animals have fewer mesenchymal progenitors at 6 months

**Fig. 6:**
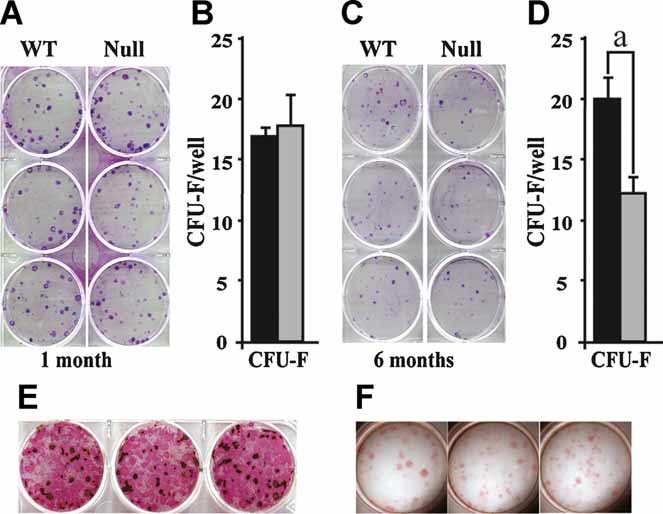
*Wnt10b*-null animals have reduced numbers of bone-derived mesenchymal progenitors. Primary bone marrow stromal cells (PBMSCs) were isolated from WT and *Wnt10b*-null animals at 1 (*A, B*) and 6 (*C, D*) months of age, grown in Mesencult medium for 7 days, and stained with Geimsa for enumeration of CFU-F. Similar cultures were switched to osteogenic (*E*) or adipogenic (*F*) medium to identify foci that could differentiate into bone or fat cells. *Wnt10b*-null PBMSCs give rise to similar numbers of CFU-F colonies compared with WT at 1 month of age (*B*) but show a 40% reduction in the number of CFU-F colonies at 6 months (*D*). Following expansion of WT PBMSCs in Mesencult, colonies were refed with osteogenic differentiation medium and then costained for alkaline phosphatase activity and calcium deposition (von Kossa) to identify activation of the osteoblast program (*E*). Similar cultures were refed with adipogenic medium and subsequently stained with oil red O to identify activation of the adipogenic program (*F*). All experiments were performed in triplicate. Technical replicates from a representative biologic replicate are shown. All experiments in this figure use mice in the FVB background. Statistical significance was evaluated by Student's *t* test; *p < .05.

Since we were able to show a loss of progenitors that have adipogenic and osteogenic potential in aged *Wnt10b-*null mice, we were curious as to whether this resulted from a general loss of mesenchymal progenitors in bone marrow–derived stromal cells. Primary bone marrow stromal cells (PBMSCs) were isolated from 1- and 6-month-old WT and *Wnt10b-*null animals and grown in Mesencult medium. Mesencult is designed for quantification of mesenchymal stem cells (MSCs) by the CFU-F assay.(27) These assays showed no difference in the number of CFU-F at 1 month of age (Fig. 6*A, B*) but revealed a 40% reduction in CFU-F in *Wnt10b*-null PBMSCs (Fig. 6*C, D*) by 6 months.

In order to confirm that colonies isolated in Mesencult represent multipotent mesenchymal progenitors, PBMSCs were allowed to form colonies by isolation in Mesencult and then were switched to either osteogenic or adipogenic medium and assessed for their ability to differentiate into osteoblasts or adipocytes. Our results show that the CFU-F isolated by growth in Mesencult were able to differentiate down both osteogenic (Fig. 6*E*) and adipogenic (Fig. 6*F*) pathways, confirming that these colonies represent a form of multipotential mesenchymal progenitor.

MSCs in mice also can be analyzed by fluorescence microfluorimetry (FACS) analysis, although the markers are not as well developed for mouse MSCs as those for analysis of human MSCs. Several lines of evidence suggest that cells with a surface marker profile of lin^–^/Sca1^+^/CD34^–^/CD44^–^ or lin^–^/Sca1^+^/CD34^–^/CD44^+^ comprise a population of cells capable of giving rise to tissues of the mesenchymal stem cell lineage.(28–30) We analyzed primary bone marrow isolates for both early (Lin^–^/Sca1^+^/CD34^–^/CD44^–^) and late MSCs (Lin^–^/Sca1^+^/CD34^–^/CD44^+^) from 2-, 3-, and 6-month-old mice(31) (Supplemental Fig. 2). This analysis, though not as definitive as the CFU assays, supports the notion that young *Wnt10b-*null mice have fairly normal numbers of bone marrow–derived MSCs but that the numbers of identifiable cells are progressively reduced as the mice age.

Together these results provide support for the idea that mesenchymal progenitor populations are reduced in *Wnt10b-*null stroma, potentially indicating that mesenchymal progenitor cells differentiate prematurely and thus are not maintained at WT levels over the lifespan of the animal.

## Discussion

Wnt signaling plays a major role in specification and growth of bones, yet the individual ligands involved, as well as target cells and mechanism, are poorly understood. Canonical Wnt signaling has been shown to both stimulate self-renewal and repress osteogenic differentiation of MSCs in vitro. Exogenous addition of Wnt3a to ex vivo cell culture has been shown to inhibit osteogenesis, as evidenced by decreased alkaline phosphatase activity and expression, and to stimulate self-renewal and expansion of immature osteoprogenitors in cultured human MSCs (hMSCs).(10,11,32) Addition of canonical Wnt signaling antagonist sFRP3 to Wnt3a-treated osteogenic cultures was sufficient to suppress inhibition of differentiation and decrease proliferation.(10) Overexpression of Wnt signaling coreceptor Lrp5 in hMSCs resulted in increased proliferation and decreased osteogenesis, an effect that was enhanced in the presence of Wnt3a.(13) In a similar study, expression of dominant-negative TCF4 or constitutively active β-catenin resulted in increased proliferation and decreased differentiation of hMSCs.(14) Together these studies suggest a role for canonical Wnt signaling in inhibition of differentiation and maintenance of immature pluripotent MSCs.

Here we show that *Wnt10b*-null mice have a progressive osteopenia resulting from defects in postnatal bone homeostasis. Specifically, the loss of *Wnt10b* results in a defect in the maintenance and/or self-renewal of mesenchymal progenitor cells in bone marrow where the number of mesenchymal progenitors is reduced over the life of the animal. Enhanced Wnt signaling is a characteristic of several adult stem cell environments,(14) and loss of Wnt signaling results in loss of stem cell maintenance in skin, bone marrow, and gut, among others.(33,34) The identities of specific Wnt ligands that maintain distinct stem cell populations are largely unknown.

Our results indicate that *Wnt10b* is one of the most important ligands for maintenance of adult mesenchymal progenitor activity that can be isolated from the bone marrow stromal niche. We demonstrate that both copies of *Wnt10b* are required for normal maintenance of adult bone homeostasis and that the loss of bone in aged heterozygous animals is as severe as that for *Wnt10b*-null mice. Haploinsufficiency phenotypes for secreted growth factors are rare, and this is the first report of haploinsufficiency in bone for a secreted Wnt ligand. Haploinsufficiency of Wnt signaling pathway components GSK3β and Lef1 has been reported previously to affect bone density preferentially in female mice,(5) and *Lrp5* heterozygous mice show losses in bone mass that are not as severe as those for *Lrp5-*null mice irrespective of gender.(35) Our finding that loss of one allele of *Wnt10b* alone is sufficient to affect bone homeostasis in male mice provides important new information related to the importance of *Wnt10b* in normal adult bone homeostasis.

Postnatal bone homeostasis is a delicate balance between bone resorption by osteoclasts and bone deposition by osteoblasts. We observed that osteoclast number and activity are unaffected, indicating that bone resorption is not a major contributor to the osteopenic defect. Interestingly, osteoblast numbers also are unaffected in *Wnt10b* mutants. This result indicates that the defect in bone homeostasis occurs in a more primitive progenitor cell but that once the progenitors become osteoblasts, they posses normal properties in vivo. Several lines of evidence support a role for *Wnt10b* in the maintenance of bipotential mesenchymal progenitor cells. First, *Wnt10b*-null mice have reduced bone volume fraction and trabecular number at 2, 3, and 6 months of age and fewer mesenchymal progenitors at 6 months of age, as assessed by the CFU-F assay. Additionally, *Wnt10b*-null mice form fewer osteogenic and adipogenic colonies in in vitro CFU-Ob and CFU-A assays. The finding that *Wnt10b*-null mice at 2 and 4 weeks of age show increased bone volume fraction and trabecular number and equivalent numbers of CFU-Ob and mesenchymal progenitors is suggestive of accelerated differentiation into osteoblasts, leading to premature depletion of the pool of mesenchymal progenitor cells available for bone deposition later in life. Taken together, these data indicate a deficiency in maintenance or self-renewal of mesenchymal progenitor cells that results in depletion of this population as *Wnt10b*-null animals age. This interpretation is very different from but not irreconcilable with the results obtained by overexpression of *Wnt10b* from heterologous promoters.(19,20)

A hallmark of stem cell self-renewal is an inhibition of differentiation. We observed that *Id2*, a Wnt target gene,(36) is expressed at lower levels in *Wnt10b*-null PBMSCs. At one level, this result confirms that loss of *Wnt10b* expression is associated with a subsequent reduction in a canonical Wnt target gene. However, *Id2* downregulation was examined because it could explain, in part, the apparent enhanced osteogenic differentiation in 1-month-old mice. Expression of *Id2* has been shown previously in a variety of progenitor cells. Downregulation of *Id2* expression is critical for differentiation of lymphoprogenitor cells toward a B-cell fate,(37) and *Id2* expression also has been shown to be important for adipogenesis.(38) One possibility is that *Wnt10b-*responsive progenitors maintain pluripotency by maintaining expression of inhibitors of differentiation. While the idea that Wnt signaling contributes to the maintenance of immature mesenchymal progenitor cells through induction of an antidifferentiation program is intriguing, but further characterization of this pathway in mesenchymal progenitors is needed. Lack of a well-defined cell surface phenotype discriminating mesenchymal progenitor and mesenchymal stem cells in the mouse makes isolation and further characterization of the pathways mediating maintenance of the progenitor pool difficult to accomplish (reviewed in ref. (39)). Elucidation of the in vivo cell surface phenotype of mesenchymal stem and progenitor cells will allow for further examination of the role of *Wnt10b* in regulation of the mesenchymal stem and progenitor cell niche in future studies.

By histomorphometry, we observed reductions in percent mineralizing surface in 1-, 2-, and 6-month-old *Wnt10b*-null mice and a decreased matrix apposition rate in 6-month-old animals. Reductions in the extent of surface mineralization are consistent with a reduction in mesenchymal/osteoprogenitor number. However, the observed reduction in matrix apposition rate at 6 months does not allow us to rule out a role for *Wnt10b* in the ability of osteoprogenitors to undergo osteogenesis. Studies seeking to determine if *Wnt10b* plays a subsequent role in differentiation of osteoprogenitors are currently under way.

Defects in bone development have been associated with mutations in several components of the Wnt signaling pathway (reviewed in refs. 6 and 7). In particular, *Lrp5*-null mice show a loss of trabecular bone(35) similar to that in *Wnt10b*-null mice described here. In both *Lrp5*-null and *Wnt10b*-null mice, the loss of bone is not associated with alterations in *Runx2* expression in PBMSCs, suggesting that *Wnt10b* acts downstream of *Runx2*. *Runx2* is required for bone development and has long been considered a key regulator of osteoblast differentiation.(24–26) In contrast, we observed decreased expression of osteoblast differentiation markers alkaline phosphatase and osteocalcin in *Wnt10b*-null PBMSCs. These results could be indicative of decreases in number or function of *Runx2-*expressing multipotential mesenchymal progenitor cells.

Overexpression of *Wnt10b* in mesenchymal progenitors results in decreased adiposity in mice.(40) Interestingly, familial mutations in the human *WNT10B* locus, resulting in a loss of protein expression, implicate *WNT10B* as a potential factor in human familial obesity.(41) Studies in our laboratory to determine if adiposity is affected in *Wnt10b*-null mice have proved largely unsuccessful. This could be due to the FVB background on which these studies were created initially because FVB mice are highly resistant to diet-induced weight gain.(42) However, backcrosses of the line into C57 also have failed to reveal a pronounced increase in adiposity. Therefore, additional studies will be required to determine the effect of *Wnt10b* loss on adiposity in mice. Additionally, participants in the human study carrying the loss-of-function allele may have additional mesenchymal defects in addition to body weight gain and should be assessed for complications associated with osteopenia.

In conclusion we have demonstrated that loss of *Wnt10b* in mice results in an age-progressive osteopenia and that loss of one allele results in a fully penetrant osteopenic phenotype by 6 months of age. Additionally, the phenotype corresponds to an age-dependent loss of multipotential mesenchymal progenitors by 6 months of age. These results extend previous studies showing a role for Wnt signaling in development and postnatal bone homeostasis.(3,7,12,35,43–45) We have provided evidence that *Wnt10b* is an endogenous Wnt ligand operating in bone and that it functions to maintain mesenchymal and/or osteoblast progenitors in adult bone. Recent identification of *WNT10B* mutations in human pedigrees of severe obesity demonstrate that altered activity at this locus contributes to additional defects in mesenchymal derivatives. No other Wnt ligand has been linked to mesenchymal progenitor function in both humans and mice, making Wnt10b a critically important gene product for the analysis of mesenchymal function.
