# Supplementary material for: Wnt10b Deficiency Results in Age-Dependent Loss of Bone Mass and Progressive Reduction of Mesenchymal Progenitor Cells
Source: J Bone Miner Res. 2010 Apr 30;25(10):2138–47. doi: 10.1002/jbmr.118 (PMC3153316; doi:10.1002/jbmr.118)
Supplement: Supplementary file 1 [file jbmr0025-2138-SD1.pdf]

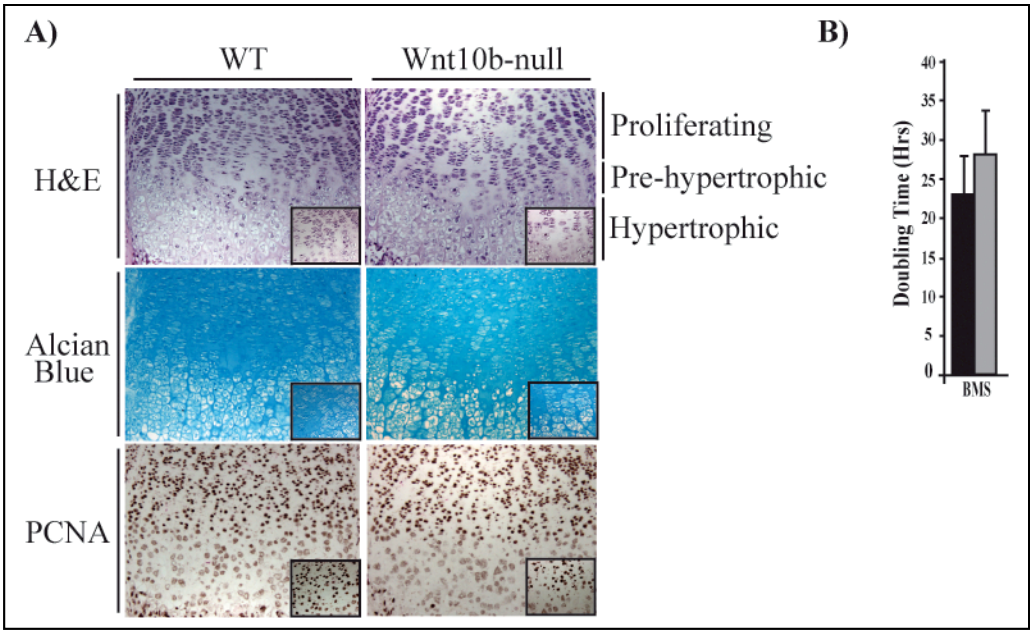


**Supplemental Figure 1: *Wnt10b*-null animals are not deficient for cell proliferation *in vivo* or *in vitro*.** (A) Femurs from post natal day four WT and *Wnt10b*-null mice were harvested, fixed in 4% PFA/PBS, paraffin embedded, and sectioned. Serial sections were stained for cellularity (H&E), cartilage (Alcian Blue) and proliferation (PCNA). Results indicate no abnormalities in growth plate formation and no difference in number of PCNA positive cells within the proliferation zone. (B) Primary bone marrow stromal cells were harvested from six month old mice, plated, allowed to expand, trypsinized, and subjected to direct cells counts by haemocytometer. Results show no difference in the rate of proliferation between WT and *Wnt10b*-null PBMSC. Experiments represent biological replicates performed in triplicate. All experiments were carried out in FVB mice.

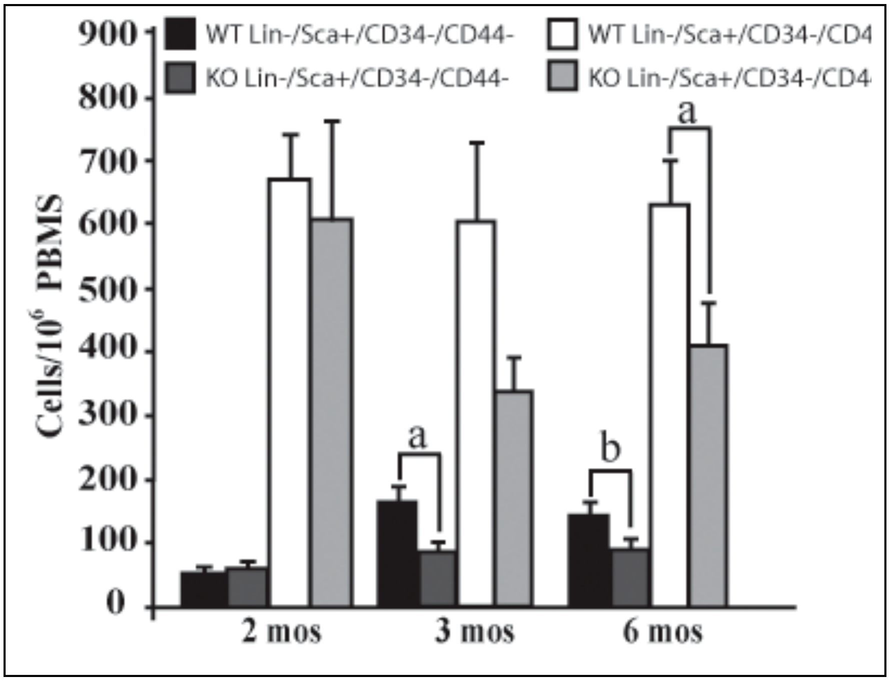


**Figure S2. Aged *Wnt10b*-null mice, but not young mice, have fewer bone-derived mesenchymal progenitors as defined by cell surface marker expression *in vivo*.** Bone marrow stromal cells (PBMSC) were isolated from WT and *Wnt10b*-null mice at two (*n=6*), three (*n=5*), and six (*n=7*) months of age and analyzed by fluorescent micro fluorimetry (FACS). Lineage negative cells were stained for expression of CD44, Sca1 and CD34 and analyzed by flow cytometry. All putative mesenchymal stem cells were defined as Lin$^-$/Sca1$^+$/CD34$^-$/CD44$^-$ or Lin$^-$/Sca1$^+$/CD34$^-$/CD44$^+$ and did not express linage markers CD45R/B220, TER119, CD8, CD4, CD3, CD11b or GR.1. No difference is observed in either population at two months of age. At three and six months of age statistically significant decreases are observe in the CD44$^-$ population of *Wnt10b*-null stromal cell isolates. Decreases in the CD44$^+$ population approach statistical significance at three months of age with statistically significant decreases apparent by six months. Statistical significance was evaluated by students t-test; $^{a}p < 0.05$. ; $^{b}p < 0.01$.

**Supplemental Table S1: Genotyping PCR Primers**

| *Primer* | *Gene ID* | *Template* | *Sequence* | *Orientation*[3] |
|---|---|---|---|---|
| *neof* | | Genomic | 5'-CATCGCCTTCTATCGCCTTCTTGA-3' | S |
| *neor* | | | 5'-GAGCTGGCCCTTAATTTGGTTTTG-3' | AS |
| *w10bEx5* | *22410* | *Genomic* | *5'-CATAACAGCACCAGTGGAAACGACAGTG-3'* | *S* |
| *w10bIN4* | | | *5'-CTGCCTTGCCTGACAGACTTTAGGACAT-3'* | *AS* |

**Supplemental Table S2: Quantitative Real Time PCR Primers**

| *Primer*[1] | *Gene ID* | *Template* | *Sequence*[2] | *Orientation*[3] |
|---|---|---|---|---|
| *Alk Phos* | 11647 | cDNA | 5'-AAACCCAGAACACAAGCATTCC-3' | S |
| | | | 5'-TCCACCAGCAAGAAGAAGCC-3' | AS |
| *Oscn* | 12096 | cDNA | 5'-TCTCTCTGACCTCACAGATGCC-3' | S |
| | | | 5'-TACCTTATTGCCCTCCTGCTTG-3' | AS |
| *Runx2* | 12393 | cDNA | 5'-CCGCACGACAACCGCACCAT-3' | S |
| | | | 5'-CGCTCCGGCCCACAAATCTC-3' | AS |
| *Wnt10b* | 22410 | cDNA | 5'-GCACCACAGCGCCATCCTCA-3' | S |
| | | | 5'-TTGCTCACCACTACCCTTCCATCC-3' | AS |
| *Id2* | 15902 | cDNA | 5'-CAGCATCCCCCAGAACAAGA-3' | S |
| | | | 5'-GCGATCTGCAGGTCCAAGAT-3' | AS |
| *ß-Actin* | 11461 | cDNA | 5'-AGGTGTGCACCTTTTATTGGTCTCAA-3' | S |
| | | | 5'-TGTATGAAGGCTTTGGTCTCCCT-3' | AS |

[1]Mouse gene targets are listed according to nomenclature standards.
[2]The nucleotide sequence of each primer is listed. All reactions required primer pairs.
[3]The orientation of the listed primer: Sense (S) or Antisense (AS).

## Supplemental Materials and Methods

### *Histology and Immunohistochemistry*

Femurs from post natal day four WT (*n=6*) and *Wnt10b*-null (*n=6*) mice were harvested, fixed in 4% paraformaldehyde/PBS, acid decalcified, and embedded in paraffin. 5μM serial sections were stained with Haemotoxilin and Eosin (H&E), or Alcian Blue following standard protocols. Immunohistochemistry for proliferating cell nuclear antigen (PCNA) was carried out on serial sections prepared following standard protocols and PCNA was detected with a mouse monoclonal antibody (Cell Signaling Technologies; PC10 mAB #2586) followed by detection with anti mouse IgG-HRP, and DAB reaction. Sections were imaged by light microscopy at 250x and 400x magnification.

## Fluorescence micro fluorimetry (FACS)

Bone marrow stromal cells were isolated from WT and *Wnt10b*-null mice at two (*n*=*6*), three (*n*=*5*), and six (*n*=*7*) months of age. Bone marrow suspension was carried out by flushing of femurs with $Ca^{2+}$-free and $Mg^{2+}$-free PBS usually yielding 30-40 X $10^6$ cells. Single-cell suspensions were depleted of erythrocytes using Tris-ammonium chloride, pH 7.2, before the incubation with anti-CD16/CD32 reducing non-specific labeling in FACS buffer (PBS, 2% FBS, 0.1% NMS). Subsequently cells were washed incubated with a combination of cell surface determinants using Mouse Hematopoietic Linage Flow Panel (eBiosciences: Biotin anti-mouse): CD3 (145-2C11), CD45R/B220 (RA3-6B2), CD11b (M1/70), erythrocyte (Ter-119) and Ly-6G (RB6-8C5). Biotinylated cells were visualized by incubating in PE-conjugated or PEcy7-conjugated stretavidin. Antibodies were obtained from BD Pharmigen or eBiosciences conjugated to

fluorescein isothiocyanate, phycoerythrin, phycoerythrin-Cy7, tricolor, allophycocyanin or allophycocyanin-Alexa Fluor 750: CD44 (Pgp-1/Ly-24), Ly-6A/E (Sca1) and CD34 (RAM34). Optimum working dilution conditions for each antibody was conducted with the appropriate isotype controls. All incubations were for 30-40 minutes at 4 ºC. After the washing, up to 1-2 X $10^6$ live or subsequently fixed with 1% paraformaldehyde were acquired with Diva Software (Becton Dickinson) or FLOWJO on a FACScan III, FACSCalibur and FACSAria (all BD Biosciences). All putative mesenchymal stem cells were defined as $Lin^-Sca1^+CD34^-CD44^-$, or $Lin^-Sca1^+CD34^-CD44^+$. Neither population expressed linage markers CD45R/B220, TER119, CD8, CD4, CD3, CD11b or GR.1.

## <u>PCR conditions</u>

### *Genotyping PCR*

Genomic DNA was harvested from tail snip biopsies following standard protocols and purified DNA was mixed with a PCR master mix containing 50 pmoles of one primer pair and PCR buffer. Neomycin PCR samples were supplemented with 0.5M betaine to improve performance. All samples were subjected to 30 rounds of polymerase chain reaction using the following conditions: (1) 95 ºC for 10 min, (2) 95 ºC for 45 seconds, (3) 57 ºC for 45 seconds, (4) 72 ºC for 1 minute, (5) return to step 2 thirty times, (6) 72 ºC for 10 minutes. Products were analyzed by gel electrophoresis and scored the presence or absence of a 314bp neomycin product and a 600bp product from Exon 5 of the mouse *Wnt10b* gene. Amplification reactions lacking Exon 5 and containing neomycin were indicative of *Wnt10b* gene deletion.

***Quantitative rt-PCR***

cDNA, primers, and Power SYBR® green 2X Master mix (Applied Biosystems #4368706) were combined and analyzed with the DNA Engine Opticon 2 Real-Time PCR Detection System (Bio-Rad) using the following conditions: (1) 50 ºC for 2 minutes, (2) 95 ºC for 10 minutes, (3) 95 ºC for 15 seconds, (4)  60 ºC for 30 seconds, (5)  72 ºC for 30 seconds, (6) return to step 3 forty times.    Melting curves were calculated by BioRad software and reactions with C(t) < 35 cycles were scored as "expressed" and were then analyzed for single peak melting curves.
